# Bio-Morphological Reaction of Human Periodontal Ligament Fibroblasts to Different Types of Dentinal Derivates: In Vitro Study

**DOI:** 10.3390/ijms22168681

**Published:** 2021-08-12

**Authors:** Serena Bianchi, Leonardo Mancini, Diana Torge, Loredana Cristiano, Antonella Mattei, Giuseppe Varvara, Guido Macchiarelli, Enrico Marchetti, Sara Bernardi

**Affiliations:** 1 Department of Life, Health and Environmental Sciences, University of L’Aquila, 67100 L’Aquila, Italy; serena.bianchi@univaq.it (S.B.); leonardo.mancini@graduate.univaq.it (L.M.); diana.torge@graduate.univaq.it (D.T.); loredana.cristiano@univaq.it (L.C.); antonella.mattei@univaq.it (A.M.); guido.macchiarelli@univaq.it (G.M.); enrico.marchetti@univaq.it (E.M.); sara.bernardi@univaq.it (S.B.); 2 Department of Innovative Technologies in Medicine & Dentistry, University of Chieti—Pescara ‘Gabriele d’Annunzio’, via dei Vestini 11, 66100 Chieti, Italy; 3 Center of Microscopy, University of L’Aquila, 67100 L’Aquila, Italy

**Keywords:** fibroblasts, dentinal derivates, bone regeneration, dentin, autograft

## Abstract

Understanding the biological and morphological reactions of human cells towards different dentinal derivate grafting materials is fundamental for choosing the type of dentin for specific clinical situations. This study aimed to evaluate human periodontal ligament fibroblasts (hPLF) cells exposed to different dentinal derivates particles. The study design included the in vitro evaluation of mineralized dentine (SG), deproteinized and demineralized dentine (DDP), and demineralized dentine (TT) as test materials and of deproteinized bovine bone (BIOS) as the positive control material. The materials were kept with the hPLF cell line, and the evaluations were made after 24 h, 72 h, and 7 days of in vitro culture. The evaluated outcomes were proliferation by using XTT assays, the morphological characteristics by light microscopy (LM) and by the use of scanning electron microscopy (SEM), and adhesion by using confocal microscopy (CLSM). Overall, the experimental materials induced a positive response of the hPLFs in terms of proliferation and adhesion. The XTT assay showed the TT, and the SG induced significant growth compared to the negative control at 7 days follow-up. The morphological data supported the XTT assay: the LM observations showed the presence of densely packed cells with a modified shape; the SEM observations allowed the assessment of how fibroblasts exposed to DDP and TT presented cytoplasmatic extensions; and SG and BIOS also presented the thickening of the cellular membrane. The CLMS observations showed the expression of the proliferative marker, as well as and the expression of cytoskeletal elements involved in the adhesion process. In particular, the vinculin and integrin signals were stronger at 72 h, while the actin signal remained constantly expressed in all the follow-up of the sample exposed to SG material. The integrin signal was stronger at 72 h, and the vinculin and actin signals were stronger at 7 days follow-up in the sample exposed to DDP material. The vinculin and integrin signals were stronger at 72 h follow-up in the sample exposed to TT material; vinculin and integrin signals appear stronger at 24 h follow-up in the sample exposed to BIOS material. These data confirmed how dentinal derivates present satisfying biocompatibility and high conductivity and inductivity properties fundamental in the regenerative processes. Furthermore, the knowledge of the effects of the dentin’s degree of mineralization on cellular behavior will help clinicians choose the type of dentine derivates material according to the required clinical situation.

## 1. Introduction

The inflammatory and traumatic diseases occurring in the jawbones might result in a loss of the tridimensional volume of the periodontal tissues of the tooth [1]. Consequently, the periodontal ligament adhesion to the alveolar bone can decrease with mobility or dental exfoliation [2,3]. After dental extraction, the residual alveolar bone crest proceeds to a resorption process, which in some cases cannot permit the implant placement necessary to rehabilitate the created edentulism with a fixed prosthetic device [4,5].

For all these reasons, regenerative surgical techniques have been proposed and used by relying on the three principles of regeneration: cell conduction, cell induction, and cell proliferation [6]. Therefore, grafting biomaterials have been developed during the years, which could fulfill at least two of the properties of the principle of regenerations [7].

Currently, the only grafting material which fulfills the three principles of the regenerations is the autologous bone, where the structure of the bone represents the osteoconductive property, the vascular supply from the retrieved site provides the growth factors with osteoinductive properties, and the osteoblasts play a key role in the osteogenesis [8].

The need for grafting materials in bone regenerative medicine, not only in dental procedures but also in orthopedics procedures [9], pushed the research and developments (R&D) units of private and public companies to project and design several types of biomaterials from different sources. Tampieri et al., developed the so-called “green-bone” by using “native wood” as the source [10]; the coral shell, due to the osteoconductive properties, is currently studied due to its mineral architecture with osteoconduction properties [11].

Clinically, the most used grafting materials are derived from animal sources, such as bovine, equine, and swine, all with a particular grade of promising results [12].

However, the heterologous nature of the materials and the fact that the materials possess the quality of being solely osteoconductive pushed researchers to look for biomaterials that also possess osteoinductive properties, such as blood derived products of the following: platelets-rich-plasma (PRP) [13], platelets-rich-fibrin (PRF) [14], and concentrated-growth factors (CGF) [15]. These present product differences in fibrin consistency, growth factors, and methods of preparations [16]. However, their influence on the regeneration process has been positive and worthy of consideration [13,14,15,16].

The products from the blood centrifugations are widely used, either alone or in combination with the heterologous grafting materials due to the contents of the growth factors [17].

Another material with an autologous nature and which has been considered in the last years as a possible grafting material is the other available mineralized tissue: the dentine [18].

Technological innovation allowed the development of devices that transform the body of the tooth to a grafting material made of dentin [19,20,21].

Dentin is a mineralized tissue underneath the enamel, composed of 65% of inorganic material (hydroxyapatite) and 35% of organic material (collagen proteins and bone morphogenetic protein-BMP) [22].

Both these components have been found to play a key role in the osteoconductive and osteoinductive processes [21,23].

These types of materials have been tested in recent years in in vitro and in vivo experimental studies. However, controversial data are available in the literature due to the availability of dentin with different mineralization degrees [24,25,26].

The different compositions of the used dentin imply a different use of the material according to the required situation: if a simple scaffold is required, mineral dentine can be used [25]. If, instead, BMP is required to induce cellular migration and differentiation, demineralized dentin would be more required [25].

The dentin as grafting material has been reported in cases for alveolar socket preservation or bone defect correction and periodontal defect regeneration [27]. Even though the available cases report successful integration of the material, the cellular behaviors towards the dentinal derivates and the eventual differences among the several available materials are not fully investigated. The study aims to assess the biological and morphological reactions of human periodontal ligament fibroblasts (hPLFs) exposed to the presence of different types of dentine derivates.

## 2. Results

The combined analyses of XTT and the morphological observations allowed a full and integrated overview of the performance of the examined dentin derivates material in contact with the hPLFs.

### 2.1. Cell Proliferation Assays and Statistical Analysis

**Figure 1:**
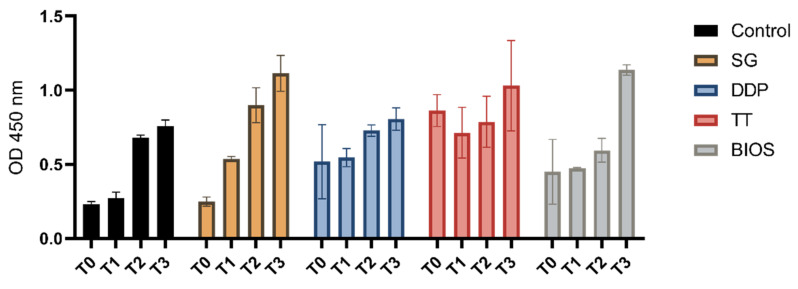
Separated graph bar showing the grouped data of mean and SD of the optical density values (*Y*-axis) of the cell not exposed to materials (Control), the cell exposed to tested materials (SG, DDP, and TT), and the cell exposed to the positive control material (BIOS) at the different timings (T0—moment of seeding; T1—24 h after seeding; T2—72 h after seeding; and T3—7 days after seeding).

As shown in Figure 1, the proliferation assays showed a proliferation growth curve in the tested dentinal grafts (SG, DDP, and TT), in the positive control material (BIOS), and in the negative control (no material).

The two-way ANOVA tests considering the variation of the OD from the moment of seeding (T0) and the other follow-up (T1, T2, and T3) were statistically significant. The post-hoc Dunnett’s analysis showed a significant variation of the growth in the T2 and T3 follow-up in the negative control. In the positive control BIOS and on the DDP experimental group, there was a significant variation at the T3 follow-up. There was significant variation at the T1, T2, and T3 in the SG experimental group, while in the TT experimental group, a statistically significant variation between T0 and the T1, T2, and T3 (Table 1) was not observed.

The two-way ANOVA tests considering the variation of the OD between the experimental groups and the negative control at the different times of seeding (T0) (to assess any difference absorbance differences of the considered materials) and the other follow-ups (T1, T2, and T3) were statistically significant. The post-hoc Dunnett’s analysis showed a significant variation of the growth at the T1 follow up between the negative control group and the experiment groups TT and DDP and a significant growth variation at the T3 follow-up between the negative control group and the experimental groups’ SG, TT, and BIOS (Table 2).

The post-hoc Bonferroni analysis showed a significant variation in the T3 follow-up between the DDP and BIOS groups (Table 3).

### 2.2. Morphological Analysis—LM

**Figure 2:**
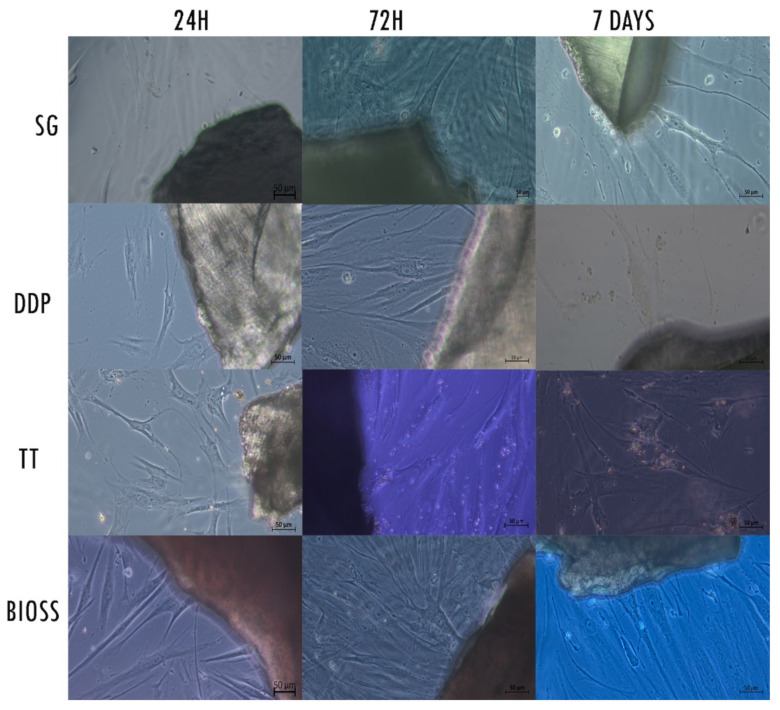
Contrast-phase light microscopy images of hPLF cells with the examined materials and the negative control at the different examined times. Magnification 20×.

The LM analysis showed a layer of healthy fibroblasts in the cells exposed to the dentinal materials and the bone material used as control, with morphological differences between them (Figure 2). At 24 h of culture, the cells exposed to the DDP and TT dentinal material appeared larger and with a polygonal shape; the cells exposed to the SG and to positive control BIOS were large but presented a more fusiform shape if compared with the DDP and TT groups. All the cells presented cytoplasmatic extensions in the proximity of the biomaterial. At 72 h, the cultures of all the samples appeared to be of higher density. The cells’ shape of all samples was large and polygonal, with a high presence of cytoplasmatic processes in the proximity of the material. The cells of the TT group presented small white particles inside the body. At 7 days, the cells of the DDP group continued to present a large and polygonal shape as well as the cells of the TT group, which also continued to present small white particles inside the body. Fibroblasts of SG and BIOS appeared polygonal with a more fusiform morphology.

### 2.3. Morphological Analysis—SEM

**Figure 3:**
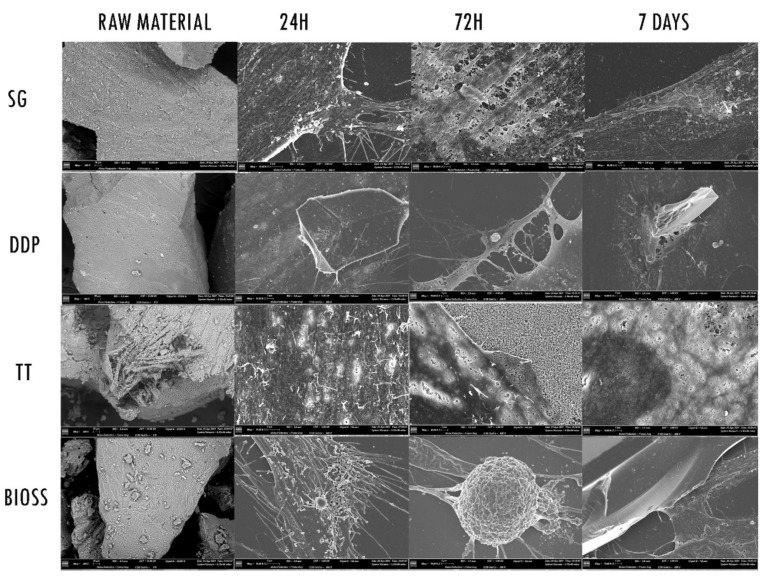
SEM images. The first column on the right shows the SEM of the different Raw Materials. Magnification 200×. The other columns show the different reaction of the fibroblasts when exposed to the experimental materials SG, DDP, and TT and the positive control BIOS, at 24 h, 72 h and 7 days. Magnification 1000×.

An initial SEM observation of the raw material highlighted the morphological differences between the tested dentinal materials and the positive control (Figure 3). In particular, the peculiar morphology of the dentinal materials was appreciable. The external morphology of SG and DDP appeared to be overlapping, with the presence of the dentinal tubules. Since DDP was submitted to the demineralization protocol, the surface appeared smoother than the SG. The morphology of the TT sample appeared more irregular and jagged; the machine ground the dentin, and it was possible to observe the internal portion of tubules. The BIOS sample appeared with an irregular surface of mineralized bone.

Regarding the fibroblasts’ behavior, the cells body exposed to the experimental biomaterials (SG, DDP, and TT) at 24 h appeared to be enlarged and with cytoplasmatic extensions. The surface of cells exposed to SG and TT, in addition, presented digitation on the membrane. The fibroblasts exposed to DDP material appeared flat covering the biomaterial but with no external cellular reaction. The cells in contact with the positive control BIOS presented several cytoplasmatic extensions on the membrane’s surface. At 72 h, it was possible to observe the different reactions of the cells exposed to experimental biomaterials. The membrane surface of the fibroblasts exposed to SG and BIOS appeared to be thicker with cytoplasmatic eversions, while the fibroblasts membrane exposed to DDP and TT appeared flat, with cytoplasmatic projections toward the biomaterials. The surface of the cells exposed to TT showed tiny holes. At 7 days, the surface morphology of the cells exposed to SG and BIOS materials continued to appear highly dynamic, with progressive thickening and with the presence of cytoplasmatic digitations likely attributable to lamellipodia and filopodia. The morphology of the surface of fibroblasts exposed to DDP and TT continues to be flattened, with cytoplasmatic extension toward the biomaterials. The membrane surface of fibroblasts exposed to TT continues to present tiny holes.

### 2.4. Morphological Analysis—CLSM

**Figure 4:**
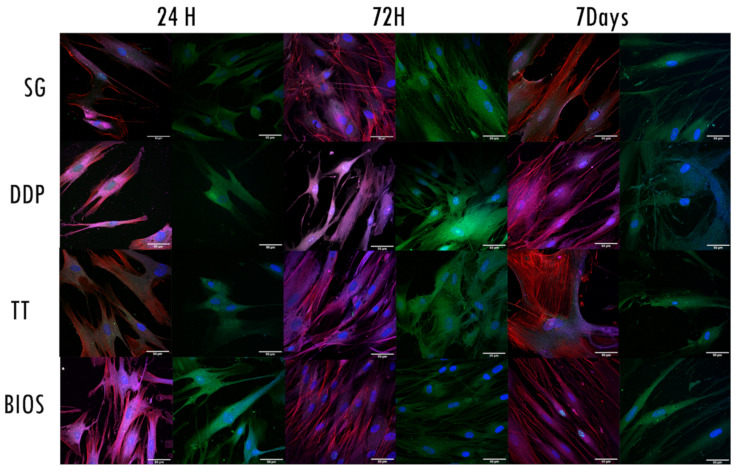
Confocal laser scanning microscopy (CLSM) images. Each row represents the exposure of the fibroblast to the different biomaterials. For each follow-up, two columns with different fluorescence stains are shown: The first one from the left shows the expression of proliferation (green signal-anti-Ki67) and the expression of actin filaments (red signal-phalloidin), vinculin (magenta signal- vinculin), and the nuclei (blue signal-DAPI). The second stain shows integrin expression (green signal-integrin αVβ3). Nuclei are stained with DAPI (blue signal). Magnification at 63×.

The CLSM observation allowed us to assess the status of the nuclei, the expression of cytoskeleton elements, such as actin, vinculin, and integrins, and the proliferative state of the cells (Figure 4). The nuclei showed an oval or rounded (blue) shape and were well represented in all samples. The proliferation marker was present in all samples and in all follow-ups.

Regarding the expressions of the cytoskeleton proteins, the fibroblasts exposed to SG material expressed vinculin integrin and actin in all the follow-ups. The vinculin and integrin signals were stronger at 72 h, whilst the actin signal remained constantly expressed in all the follow-up and well distributed in the cytoplasmatic projections.

The fibroblasts exposed to DDP material expressed vinculin integrin and actin in all the follow-ups. The integrin signal was stronger at 72 h. The vinculin and actin signals were stronger at 7 days follow-up. The actin filaments were distributed into cytoplasmatic projections.

The fibroblasts exposed to TT material expressed vinculin integrin and actin in all the follow-ups. The actin signal was stronger at 24 h and 7 days follow-ups, distributed in the cellular projections and in the cellular body. The vinculin and integrin signals were stronger at 72 h follow-up. The fibroblasts exposed to BIOS material expressed vinculin integrin and actin in all the follow-ups. The vinculin and integrin signals were stronger at 24 h follow-up. The actin signal was stronger at 72 h and 7 days follow-ups, mainly distributed along with the cytoplasmatic projections.

## 3. Discussion

### 3.1. Different Degrees of Dentinal Mineralization Stimulate Different Cellular Reactions

Dentinal derivates represent a new class of autologous biomaterial with the potential for regeneration. The development of different protocols in order to obtain a granular material with similar characteristics compared to the bone tissue allowed obtaining an autologous material that is much more easily available than the autologous bone. The available machines allow obtaining a mineralized deproteinized dentin and demineralized dentine. Both types of dentinal derivates have a solid 3D structure; in addition, demineralized dentin is also accompanied by collagen and Bone Morphogenetic Proteins (BMPs) [28].

The idea of using the mineral part of the body without performing a bone auto-transplant is not new, however, the use and commercialization of the machines that process the tooth are recent developments, and not all reactions of the oral hard tissues relative to these grafts have been clarified [29,30].

Of note, the processing methods cannot overcome quality issues of the dentinal tissue source. A clean surface obtained with accurate root scaling and debridement [31] or by using laser therapy or photodynamic therapy for selective biofilm removal [32] promotes fibroblast adherence [31]. Therefore, a pre-treatment of the tooth source would increase the quality of obtained graft. Dentinal derivates have been used to correct bone defects prior to the implant placements and for the alveolar socket preservations.

Mazor et al. [33] assessed the dynamic reaction of post-extractive sites histologically, successively hosting implants filled with mineralized dentin derivates. In particular, it was possible to assess an alveolar crest as dimensionally and qualitatively stable; histological analysis showed the presence of newly formed bone tissue and the remainder of particle dentin [33]. Implants remained osseointegrated at the one-year follow-up [33].

Santos et al. [34] conducted a clinical trial comparing the integration of mineralized dentin and xenograft in post-extractive sites for ridge preservation and implant placements. The histological analysis revealed a quantity of newly formed bone in the sites filled with the dentin and no differences in the implant therapy outcomes [34].

In their case series, Minetti et al. [35] histologically compared the demineralized dentine graft obtained with the same protocol as we used for TT material, placed in post-extractive sites with a graft mixing the demineralized dentine and the mineralized deproteinized bone. At the 4 months follow-up, the histological biopsies showed a higher percentage of newly formed bone in the sites grafted with the dentine than the sites grafted with the mixed materials [35].

The data from our in vitro study revealed a good response from the hLPFs to all the materials, with similar behavior in cases of use of mineralized dentin (SG) and deproteinized mineralized bovine bone (BIOS) and different reactions to the two types of demineralized dentin (DDP and TT). In terms of surfaces reactions, the mineralized materials similarly induced a thickening of the membrane, which likely can be related to the mineral content. In terms of the timing of response, the TT gave the best results considering the proliferation and adhesions at the T2, probably due to the proteins and induction factors missing in the DDP, although the cells showed a later response (T3) to the demineralized scaffold.

The low number of the patient providing the dentinal material and, consequently, the lack of experimental repeats limit the strength of the obtained results. However, these limitations regarding sources of variation that we did not consider (age and gender of patients) can represent a further field of research to expand the body of knowledge relative to the efficacy of the dentinal materials as a graft.

### 3.2. Different Mineralization Degrees Affect Fibroblast Proliferation

In our study, the different types of dentinal derivates induced different types of biological and morphological behavior. All the experimental materials induced a significant proliferation in the hPLFs. Interestingly the mineralized deproteinized dentin SG induced a significant growth difference at all the follow-up times, and at the T3 follow-up, all the materials, except for the DDP, induced a significant growth compared with the negative control.

These data are consistent with the literature: as firstly reported by Yeomans et al. [36], the demineralized dentin placed in bone sites induced the formation of bone earlier than the mineralized dentin. Bessho et al. [37] assessed the presence of BMPs in the demineralized dentin, which represents the factors of the cellular induction. This has been also confirmed by Blum et al. [38], who reported that the demineralization of the dentin facilitates the release of the cellular induction. Demineralized dentin, also saving the collagen matrix, provides a suitable carrier for inductive growth factors; mineralized materials, instead, take longer to degrade, and the mineral content influences the cellular activity. Our data are in line with those findings reported in the literature [39,40]. As reported by Alliot-Licht et al. [41], the presence of hydroxyapatite causes a first initial delay in the proliferation activity of the hPLFs and an increase on the third day of in vitro culture.

### 3.3. Different Morphological Fibroblast Reaction toward Different Type of Dentinal Graft

The LM and SEM observations showed how the dentinal derivates induced critical morphological changes.

In all experimental settings, the observed changes in the shape of the fibroblasts suggest a positive reaction to the material exposure in terms of cellular activation, as confirmed by the dimensional increase in the cell body, the polygonal shape morphology, and the development of several cellular digitations towards the material granules.

In addition, the SEM images allowed us to assess the fibroblast membrane reaction to the different types of dentine derivates and the positive control. It was interesting to note how the fibroblasts reacted by thickening their membrane in the presence of the mineralized materials (SG and BIOS). By contrast, the fibroblasts in contact with demineralized dentin (DDP and TT) reacted by flattening their membrane. These findings are consistent with those in the literature. Tabatabaei et al. [42] reported how the deproteinized mineralized dentin induced elongation of human dental pulp stem cells adhering on the graft surface. Bono et al. [28] found that the demineralized dentin induced adhesion of osteoblasts on the surfaces of the tested graft. However, the authors did not observe a thickening of the membrane [28].

CLSM observation strongly supported the LM and SEM observations. The progressive increase and the dynamic expression and distribution of vinculin, actin, and integrin indicate the cytoskeletal reaction to the experimental material.

Even though these cytoskeletal markers were expressed in all experimental and control materials, there were differences depending on the follow-up times. At 24 h, the actin filaments were visible in all materials. Vinculin and integrin are mostly expressed in the DDP and BIOS material, signifying an earlier adhesion of the fibroblasts [43,44]. At 72 h, the demineralized dentin DDP and TT showed stronger vinculin and integrin signals than SG and BIOS, which presented a well-defined actin expression, confirming the reorganization of the cytoskeletal components. Actin is a cytoskeletal protein accountable for the morphological changes of the cells and for the movement of cellular organelles; therefore, it is a signal of the activation of fibroblasts [45,46,47]. At the 7 day follow-up, vinculin was highly expressed in the fibroblasts exposed to the DDP material, whilst actin expression was more notable in fibroblasts exposed to the other materials. Our results are consistent with the ones in the literature: Hakkinen et al. [48] showed different fibroblast reactions in terms of migration and adhesion in 2D cultures, underlining how actin plays a key role in the cellular migration and adhesion to the extracellular matrix.

From these data, it appears that the mineralization degree and the protein content influence the hPLF reactions in terms of proliferation, adhesion, and migration. The DDP, which provides a demineralized scaffold, promoted early adhesion of the cells. On the other hand, the SG, which provides a solid mineral scaffold, resulted in the adhesion protein expression at the middle point (72 h) similarly to the BIOS and to the TT, which provides a demineralized scaffold with BMPs releases. Interestingly, the DDP showed a late adhesion response of the fibroblasts at 7 days follow-up.

## 4. Materials and Methods

### 4.1. Samples: Sampling Procedures and Dentine Derivates Preparation

Four wisdom teeth were previously extracted due to surgical reasons. The samples were kindly provided from a healthy, 25 year old, and non-smoking Italian woman after obtaining her written informed consent according to the Helsinki Declaration on the Ethical Principles for Medical Research Involving Human Subjects. The teeth were stored in physiological solutions that were used for dentine processing according to three different protocols:

- Tooth Transformer (TT Tooth Transformer S.r.l., Milan, Italy)—the derived dentine will be named in the manuscript as TT;
- Smart dentine Grinder (KometaBio Inc., Cresskill, NJ, USA)—the derived dentine will be named in the manuscript as SG;
- Smart dentine grinder within house protocol—the derived dentine will be named in the manuscript as DDP.

Before starting, all molars were cleaned from residual periodontal ligaments fibers and residual calculus by using a piezoelectric instrumentation Piezon (EMS Electro Medical Systems SA, Nyon, Switzerland). All the teeth were reduced into small pieces with a diamond torpedo bur # 0.12 (Komet Italia S.r.l., Milano, Italy) in order to permit mincing.

#### 4.1.1. Tooth Transformer Protocol

The extracted tooth was completely processed in the mill with pre-calibrated blades and chemical solutions. After 25 five minutes of processing, the dentine was ready to be used in cells culture. The particles size obtained, according to the manufacturer’s instructions, is Ø < 1 mm [26]. The solutions used included 6 reagents: (I) demineralization reagent, (II) and (III) washing solutions, (IV) sterilization reagent, and (V) and (VI) washing agents.

#### 4.1.2. The Smart Dentine Grinder Protocol

**Figure 5:**
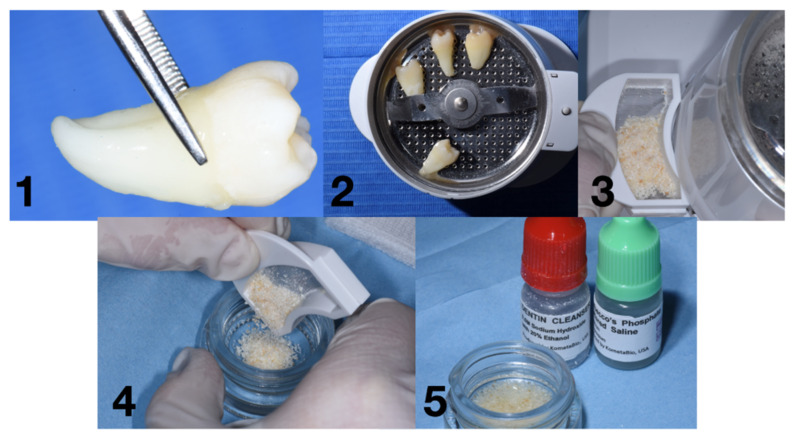
Workflow for the Smart Grinder protocol: (**1**) removal of tartar and periodontal ligament Figure (**2**). milling process, (**3**) particles ready to be chemically treated, (**4**) particles in the mixing dish, and (**5**) particles after the chemical process.

The protocol consists of two solutions used after the milling process. Particles of Ø 0.25 to 1 mm were obtained. The chemical solutions are (I) a dentine cleaner (0.5 mL Sodium Hydroxide with Ethanol) and (II) Dulbecco’s Phosphate Buffered solution. After the milling process, the particles were stored in a sterile container (mixing dish), and the dentine cleanser was used in order to cover the entire particles for 5 min. After this first phase, the cleanser was removed, and the buffered solutions were added to clean the residues. This step, according to the manufacturer’s instructions, was applied two times (Figure 5).

#### 4.1.3. The Smart Dentine Grinder Protocol Internally Modified

The Smart Dentine Grinder internally modified protocol consists of the replacement of the dentine detergent and demineralizer (0.5 mL sodium hydroxide with ethanol) with 0.5 mL of nitric acid 2% in order to demineralize the dentin. The conditioning time lasted 5 min.

#### 4.1.4. Control Material

As control material, a demineralized and deproteinized bovine bone, commonly used in dental surgical procedures (Geistlich Bio-Oss^®^, Granules 0.25–1 mm), was used.

### 4.2. Cell Culture

The cultures of hPLFs cell line from ScienCell Research Laboratories were conducted according to the manufacturer’s instructions. The initial vial containing 5 × 105 cells in 1 mL of volume was incubated under standard cell culture conditions (37 °C in 5% CO_2_) and seeded in three plastic culture dishes in Fibroblast Medium containing the following: 500 mL of basal medium, 10 mL of fetal bovine serum (FBS), 5 mL of fibroblast growth supplement, and 5 mL of penicillin/streptomycin solution (10,000 IU/mL of Penicillin; 10 µg/mL Streptomycin). At the sub-confluence stage, the detachment stage followed using 0.05% trypsin, and subcultures with the density of 110 cells/mm^2^ were initiated. The cells used for all experimental assays came from the subculture passages 8.

### 4.3. Cell Proliferation Assays and Statistical Analysis

The cells were incubated in 96 well plates under standard cell culture conditions. Two micrograms of the tested material were added to the 96 well microplates. At the moment of seeding (T0) and at 24 h (T1), 72 h (T2), and 7 days (T3) follow-up, the XTT Assay (Cayman Chemical, Ann Arbor, MI, USA) was used to assess cellular proliferation activity at a read absorbance wavelength of 450 nm. The XTT tests were performed with three technical replicates.

After assessing the normal distribution of the data, the two-way ANOVA test and Dunnett’s post-hoc test for multiple comparisons were performed in order to assess any significant variations within the experimental groups considering the T0 and the T1, T2, and T3 follow-ups and between the experimental groups and the negative control at each follow-up. The Bonferroni post-hoc test was used to assess any significant variation between the experimental materials at each follow-up.

Statistical analysis and graphs were performed by using GraphPad Prism 9.1.1 (GraphPad Software, San Diego, CA, USA).

### 4.4. Morphological Analysis

The qualitative evaluation of cells was performed by light microscopy (LM) in order to have a first-sight overview of the culture. Then, scanning electron microscopy (SEM) and confocal laser scanning microscopy (CLSM) were used to observe the eventual morphological reactions.

#### 4.4.1. Morphological Analysis—LM

Cells were plated in 60 mm diameter plastic culture dishes with the tested and the control material and were incubated under cell culture conditions. After 24 h, 72 h, and 7 days from the seeding, the dishes were observed by using a phase-contrast light microscope (ZEISS Primovert, Jena, Germany) and the use of ZEISS Axiocam 208 color camera to capture the images at 10× and 20×.

#### 4.4.2. Morphological Analysis—SEM

After 24 h, 72 h, and 7 days from the seeding of the 60 mm diameter plastic culture dishes containing the cover glasses the test (SG, DDP, and TT) and the control (BIOS) materials, the cells were fixed by using a 2% solution of glutaraldehyde and were processed for the SEM, as described previously [18]. Briefly, the samples were processed for dehydration in ascending concentrations of ethanol solutions of 70%, 80%, and 90% and three times at 100% for 10 min each. Afterward, the samples were immersed for 3 min in 100% hexamethyldisilane (HDMS-Sigma-Aldrich S.r.l., Milan, Italy). Subsequently, the samples were air-dried by the evaporation of HDMS and placed into a desiccator for 25 min to prevent water contamination. The samples were mounted on metal stubs, gold stained, and then observed by SEM (GEMINI_SEM, Zeiss, Germany) at different magnifications by using secondary electrons and InLens probes.

#### 4.4.3. Morphological Analysis—CLSM

Cells seeded on coverslips were fixed with 4% paraformaldehyde in PBS for 10 min at RT, permeabilized with 0.1% Triton X-100 in PBS for 5 min at RT, and the nonspecific binding sites were blocked with 10% bovine serum albumin (BSA) in PBS for 10 min at RT (blocking solution). After washing, the cells were incubated with a mixture of mouse anti-Ki67 (Dako, Denmark A/S) and rabbit anti-vinculin (1:400, Immunological Sciences, distributed by Societa Italiana Chimici, Rome, Italy) primary antibodies (triple staining) or with mouse anti-Human integrin αVβ3 monoclonal antibody (single staining 1:300, Immunological Sciences) O/N at 4 °C. After the washings, the primary antibodies were revealed by a mixture of Alexa Fluor 488 anti-mouse IgG and Alexa Fluor 633 anti-rabbit IgG secondary antibodies (1:2000) and Phalloidin Alexa Fluor 546 (1:300) (triple staining) or with Alexa Fluor 488 anti-mouse IgG secondary antibody alone (Immunological Sciences) for 30 min at RT. Both primary and secondary antibodies were diluted in a blocking solution. The controls were performed by omitting the primary antibody. Coverslips were mounted with Vectashield Mounting Medium containing DAPI (Vector Laboratories, Burlingame, CA, USA) and observed at a Leica TCS SP5 confocal microscope (Leica, Mannheim, Germany). Data were acquired by Leica LAS AF software, and a minimum of 20 images for each determination was analyzed.

## 5. Conclusions

The results from the present study show how different degrees of dentinal mineralization induces different cellular behaviors in terms of proliferation, migration, and adhesion (as shown by the proliferative assays); the expression of cytoskeletal elements and the change of the external morphology (as shown by the LM and SEM observation). The knowledge of the inductive and conductive effects of the degree of mineralization of the dentine on the cellular behavior will help the clinician in the choice of the type of dentine derivates material according to the required clinical situation.

## Figures and Tables

**Table 1:** Summary of the two-way ANOVA comparison and Dunnett’s multiple comparison test considering the time factor.

Two-Way ANOVA Comparison the Different Time of Follow-Up			
Source of Variation	% of Total Variation	F (DFn ^1^; DFd ^2^)	*p* Value
**Interaction**	17.19	F (12.40) = 4.149″	0.0003
**Time**	51.85	F (3 40) = 50.04″	<0.0001
**Treatment**	17.14	F (4.40) = 12.40″	<0.0001
**Dunnett’s Analysis**	**Mean Diff.**	**95.00% CI of diff.**	***p* Value**
**Control**			
T0 vs. T1	−0.040	−0.29 to 0.21	ns
**T0 vs. T2**	−0.44	−0.70 to −0.19	0.0003
**T0 vs. T3**	−0.52	−0.77 to −0.27	<0.0001
**SG**			
**T0 vs. T1**	−0.28	−0.54 to −0.03	0.02
**T0 vs. T2**	−0.65	−0.90 to −0.39	<0.0001
**T0 vs. T3**	−0.86	−1.12 to −0.61	<0.0001
**DDP**			
T0 vs. T1	−0.02	−0.28 to 0.22	ns
T0 vs. T2	−0.21	−0.46 to 0.04	ns
**T0 vs. T3**	−0.28	−0.54 to −0.03	0.02
**TT**			
T0 vs. T1	0.14	−0.06 to 0.36	ns
T0 vs. T2	0.07	−0.13 to 0.28	ns
T0 vs. T3	−0.16	−0.37 to 0.04	ns
**BIOS**			
T0 vs. T1	−0.02	−0.23 to 0.18	ns
T0 vs. T2	−0.14	−0.35 to 0.06	ns
**T0 vs. T3**	−0.68	−0.89 to −0.47	<0.0001

^1^ Number of degrees of freedom; ^2^ degrees of freedom error.

**Table 2:** Summary of the two-way ANOVA comparison and Dunnett’s multiple comparison test considering the exposure to the different materials.

Two-Way ANOVA Comparison the Different Time of Follow-Up			
Source of Variation	% of Total Variation	F (DFn ^1^; DFd ^2^)	*p* Value
**Interaction**	17.19	F (12.40) = 4.149″	0.0003
**Time**	51.85	F (3.40) = 50.04″	<0.0001
**Treatment**	17.14	F (4.40) = 12.40″	<0.0001
**Dunnett’s Analysis**	**Mean Diff.**	**95.00% CI of diff.**	***p* Value**
**T0**			
Control vs. SG	−0.01	−0.28 to 0.24	ns
**Control vs. DDP**	−0.28	−0.55 to −0.02	0.03
**Control vs. TT**	−0.63	−0.89 to −0.36	<0.0001
Control vs. BIOS	−0.21	−0.48 to 0.04	Ns
**T1**			
Control vs. SG	−0.26	−0.52 to 0.00	ns
**Control vs. DDP**	−0.27	−0.53 to −0.00	0.04
**Control vs. TT**	−0.44	−0.70 to −0.17	0.0005
Control vs. BIOS	−0.20	−0.46 to 0.06	ns
**T2**			
Control vs. SG	−0.22	−0.48 to 0.04	ns
Control vs. DDP	−0.04	−0.31 to 0.21	ns
Control vs. TT	−0.10	−0.37 to 0.15	ns
Control vs. BIOS	0.08	−0.18 to 0.34	ns
**T3**			
**Control vs. SG**	−0.35	−0.62 to −0.09	0.0053
Control vs. DDP	−0.04	−0.31 to 0.21	ns
**Control vs. TT**	−0.27	−0.53 to −0.00	0.04
**Control vs. BIOS**	−0.37	−0.64 to −0.11	0.002

^1^ Number of degrees of freedom; ^2^ Degrees of freedom error.

**Table 3:** Bonferroni multiple comparison test considering the exposition to the different materials.

Bonferroni Analysis	Mean Diff.	95.00% CI of Diff.	*p* Value
**T1**			
SG vs. DDP	−0.01	−0.33 to 0.31	ns
SG vs. TT	−0.17	−0.50 to 0.14	ns
SG vs. BIOS	0.06	−0.26 to 0.38	ns
DDP vs. TT	−0.16	−0.49 to 0.15	ns
DDP vs. BIOS	0.07	−0.25 to 0.39	ns
TT vs. BIOS	0.24	−0.08 to 0.56	ns
**T2**			
SG vs. DDP	0.17	−0.15 to 0.49	ns
SG vs. TT	0.11	−0.21 to 0.43	ns
SG vs. BIOS	0.30	−0.02 to 0.63	ns
DDP vs. TT	−0.05	−0.38 to 0.26	ns
DDP vs. BIOS	0.13	−0.19 to 0.45	ns
TT vs. BIOS	0.19	−0.13 to 0.51	ns
**T3**			
SG vs. DDP	0.30	−0.01 to 0.63	ns
SG vs. TT	0.08	−0.24 to 0.40	ns
SG vs. BIOS	−0.02	−0.34 to 0.30	ns
DDP vs. TT	−0.22	−0.55 to 0.10	ns
**DDP vs. BIOS**	−0.33	−0.65 to −0.00	0.04
TT vs. BIOS	−0.10	−0.43 to 0.22	ns

## Data Availability

Data can be requested from the corresponding author upon reasonable request.
